# Utility of Immunohistochemistry and* ETV6* (12p13) Gene Rearrangement in Identifying Secretory Carcinoma of Salivary Gland among Previously Diagnosed Cases of Acinic Cell Carcinoma

**DOI:** 10.1155/2017/1497023

**Published:** 2017-04-06

**Authors:** Rana Naous, Shengle Zhang, Alfredo Valente, Melissa Stemmer, Kamal K. Khurana

**Affiliations:** Department of Pathology, SUNY Upstate Medical University, Syracuse, NY, USA

## Abstract

*Objective*. Secretory carcinoma is a recently described entity with characteristic immunoprofile and* ETV6* (12p13) rearrangement. Before its initial description, it was generally diagnosed as acinic cell carcinoma (ACCi). We evaluated immunoprofile and* ETV6* rearrangement in cytological and surgical cases of previously diagnosed ACCi, in an attempt to identify any misclassified SC.* Methods*. Fifteen cytology and surgical cases of ACCi diagnosed over a 13-year period were retrieved and subjected to immunohistochemistry for S-100, mammaglobin, GATA-3 and DOG-1 as well as FISH for* ETV6* (12p13).* Results*. Of the 8 cytology cases, only 1 was positive for S100, GATA-3, and mammaglobin, and negative for DOG-1. It also demonstrated* ETV6* rearrangement and was reclassified as SC. The same immunoprofile was present in 2 of the 13 surgical cases.* ETV6* rearrangement characterized by 3′ interstitial deletion was detected in one of these cases and was reclassified as SC. Immunohistochemistry and* ETV6* rearrangement were useful in identifying 2 (13.3%) cases misclassified as ACCi.* Conclusions*. Characteristic immunoprofile and* ETV6* gene rearrangement may prove useful in identifying cases of SC. The presence of* ETV6* 3′ interstitial deletion in one of our cases suggests that there may be additional* ETV6* related genetic alterations contributing to the pathogenesis of SC.

## 1. Introduction

Secretory carcinoma (SC), previously called mammary analogue secretory carcinoma, is a recently described salivary gland malignancy that bears a striking resemblance to secretory carcinoma of the breast [1]. In addition, it shares the immunophenotype and the characteristic ETV6-NTRK3 translocation of secretory breast carcinoma. Prior to its initial description as a distinctive salivary gland neoplasm, SC was erroneously misdiagnosed as adenocarcinoma (not otherwise specified), mucoepidermoid carcinoma, and especially acinic cell carcinoma (ACCi) [2, 3].

SC has been most commonly misclassified as ACCi due to overlapping histologic features in both tumor types that include cytologically bland cells with granular cytoplasm and multiple architectural patterns comprising of solid, follicular, microcystic, and papillary-cystic growth. Similar to surgical resection specimens, the cytologic distinction between ACCi, especially one that is zymogen granule poor, and SC on FNA specimen can be diagnostically challenging without molecular or immunohistochemical study [4–9].

Bishop et al. recently reported that 19% of parotid gland tumors with a prior histopathologic diagnosis of ACCi were actually SCs [4]. The purpose of this study was to evaluate the immunoprofile and* ETV6* (12p13) gene rearrangement in cytological as well as surgical cases of previously diagnosed ACCi at our institution, in an attempt to identify any misclassified SC cases.

## 2. Methods

### 2.1. Cases

All cases of salivary gland ACCi diagnosed over a 13-year period were retrieved from our cytology and surgical pathology archives at the SUNY Upstate Medical University Hospital.

### 2.2. Immunohistochemistry

Immunohistochemical studies were performed on formalin fixed and paraffin embedded cell blocks and surgical block sections of the tumor. Ventana Benchmark autostainer (Ventana Medical Systems, Inc., Tucson, AZ) was used with autostaining protocols. Automated program of the Ventana autostainer was used to carry out deparaffinization and antigen retrieval. The primary antibodies and final dilutions were S100 protein (Thermo Scientific Polyclonal, 1 : 200), GATA-3 (Biocare Medical Clone L50-823, prediluted), mammaglobin (Cell Marque Clone 304-1A5 + 31A5, prediluted), and DOG-1 (Cell Marque Clone SP31, prediluted).

### 2.3. ETV6 FISH

FISH with ETV6 break-apart probes (CytoTest, Rockville, MD; Abbott Molecular Inc., Des Plaines, IL) were performed on tissue sections (3 to 5 *μ*m). Deparaffinization was done with xylene (3 times, 5 min each) and ethanol (2 times, 1 min each) and then dried. The slides were then pretreated with boiled citrate buffer (pH 6.0) for 10 minutes followed by protease digestion for another 20 minutes. After the probes were applied, the slides were placed on a Hybrite at 73°C for 5 minutes and then at 37°C overnight with coverslips. After washing, the slides were counterstained with DAPI I and were examined using a fluorescence microscope with appropriate excitation and emission filters. FISH signals were counted on at least 40 morphologically intact, nonoverlapping nuclei. In normal cells, a 2-fusion signal (yellow) pattern was present. Cells with break-apart rearrangement showed 1 fusion (red and green overlapping), 1 green, and 1 orange signals. Cells with interstitial deletion may show 1 fusion (red and green overlapping), 1 green, or 1 orange signal. On the basis of our previous assay validation, a ratio >0.10 of break-apart or deletion signals to intact signals was considered positive.

### 2.4. Cytopathology and Histopathology

Archival cytopathologic and histopathologic material was reviewed for cytologic and histologic features of cases with and without ETV6 rearrangement.

## 3. Results

The demographics and ancillary test findings of all cytology cases and surgical cases are summarized in Tables 1 and 2, respectively. The mean age of patients was 49.3 years (range 10 to 72 years, males [5] and females [10]). Anatomic locations included left parotid (10 cases) and right parotid (5 cases).

**Figure 1:**
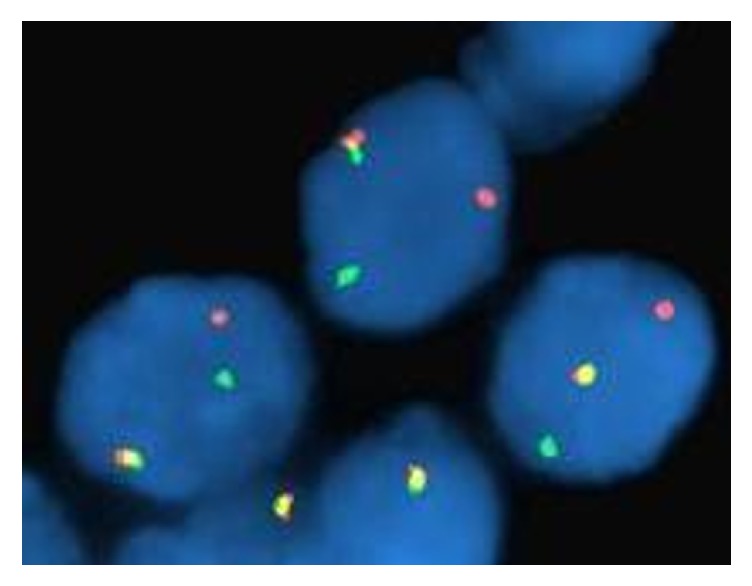
ETV6-NTRK fusion transcript (case 6) translocation seen by FISH.

Eight cytology cases with original diagnosis of ACC involving parotid gland were identified. Cell blocks were available for all of these cases. One of 8 cases (14.3%) demonstrated the ETV6 translocation by FISH (Figure 1) and remaining 7 cases were negative for translocation.

**Figure 2:**
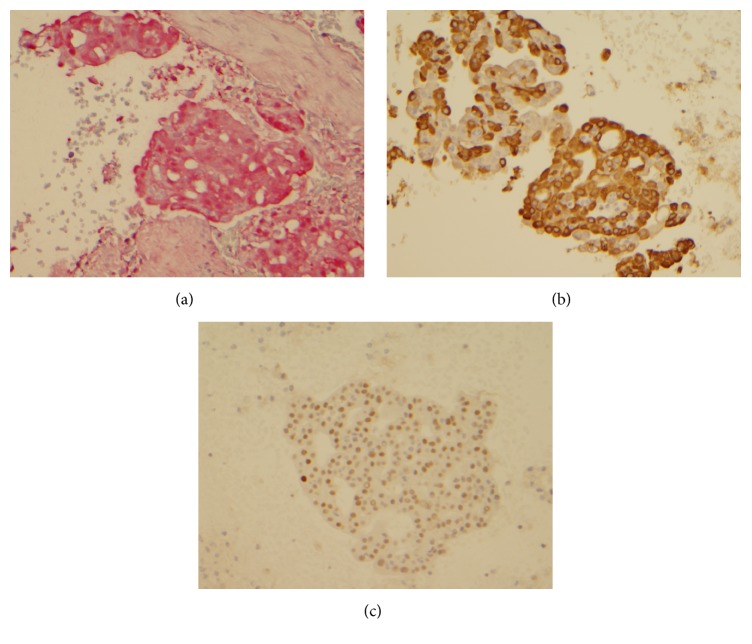
Cell block section (case 6) showing tumor cells with immunopositivity for S100 (a), mammaglobin, (b) and GATA-3 (c).

Immunohistochemistry revealed that tumor cells in translocation positive tumor were positive for S100, mammaglobin, and GATA-3 and negative for DOG-1 (Figures 2(a)–2(c)). All 7 cases with negative translocation were negative for S100, mammaglobin, and GATA-3 and DOG-1 staining was noted in only 6 cases.

**Figure 3:**
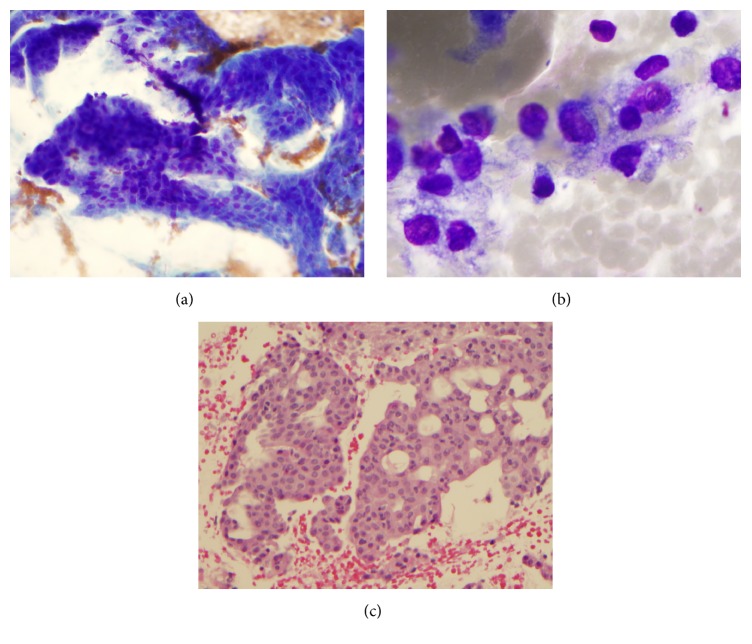
Fine needle aspiration of secretory carcinoma. (a) Arborizing papillary fragments with fibrovascular cores in a granular and cystic background (Diff-Quik Stain, ×200), (b) single cells with vacuolated cytoplasm (Diff-Quik stain ×1000), and (c) cell block showing tumor cells with prominent cribriform configuration and eosinophilic secretions (H and E, ×200).

Cytologically translocation positive tumor showed arborizing papillary fragments with fibrovascular cores (Figures 3(a)–3(c)). Single cells with characteristic vacuolated cytoplasm were identified. Cell block showed similar tumor cells with prominent cribriform configuration and eosinophilic secretions.

**Figure 4:**
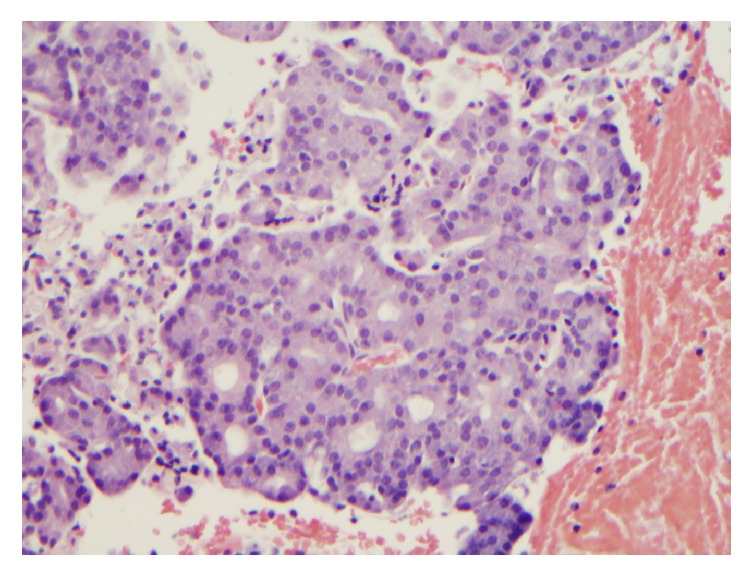
Cell block section from a case of acinic cell carcinoma exhibiting prominent cribriform configuration of tumor cells (H and E, ×200).

Seven translocation negative cases demonstrated papillary architecture with arborizing vessels 4 cases, vacuolated tumor cells (4 cases), solid sheets of bland tumor cells with eccentrically placed nuclei (7 cases), and zymogen granules (3 cases). Cribriform pattern was apparent in cell block sections of 3 cases (Figure 4). Revised cytologic diagnosis based on immunohistochemical and translocation findings was changed in only one case from ACC to SC.

Thirteen parotid gland resection specimens of cases with original diagnosis of ACCi were identified (see Table 2). Six of these cases (cases 1 to 6) had preoperative FNA aspiration cytology and corresponded with cases 1 to 6 in Table 1. Two of the 13 cases (15.38%) demonstrated ETV6 gene rearrangement characterized by 3′ interstitial deletion in one case and ETV6 translocation in the other case. The remaining 11 cases did not demonstrate any ETV6 gene rearrangement.

**Figure 5:**
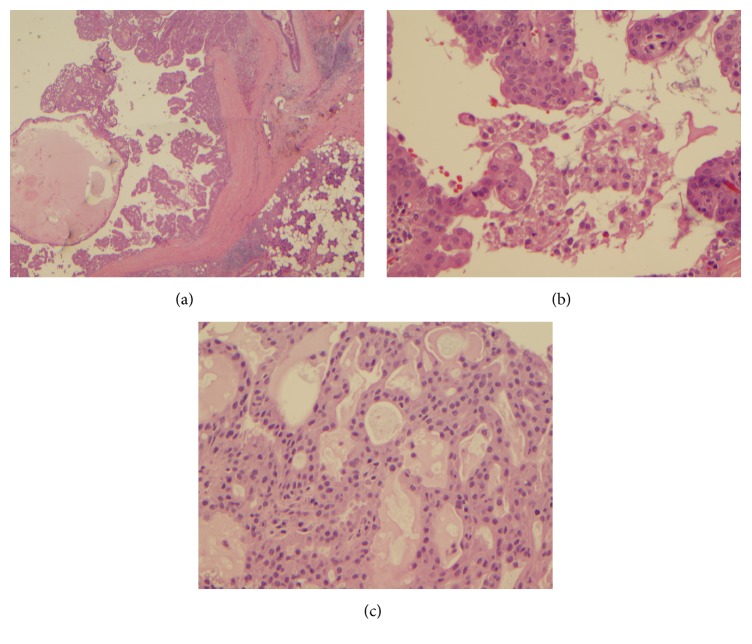
((a)–(c)) Histologic features of secretory carcinoma. (a) Tumor is well circumscribed (H and E, ×20); (b) tumor cells exhibited clear cytoplasm, were focally vacuolated, and lacked zymogen granules (H and E, ×200); (c) cribriform pattern was also noted with eosinophilic secretion in the glands (H and E, ×200).

**Figure 6:**
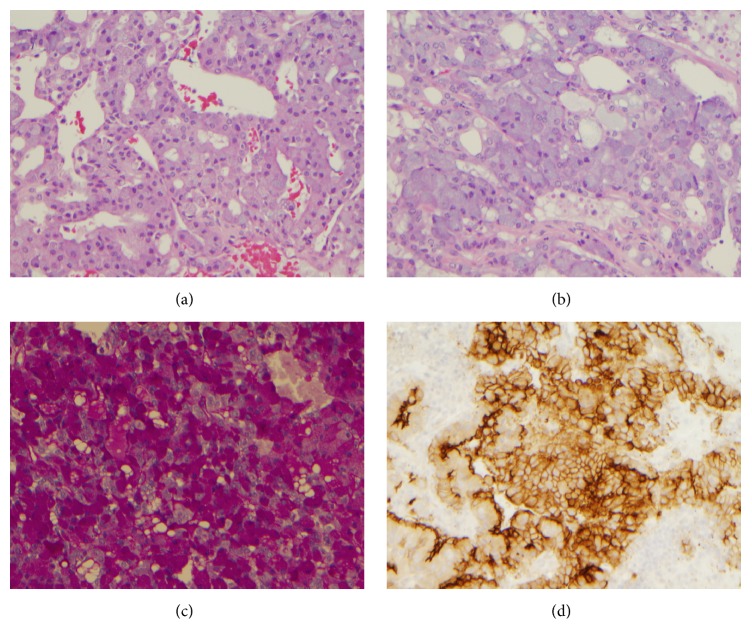
Histologic features of acinic cell carcinoma. (a) Prominent microcystic and papillary pattern (H and E, ×200); (b) tumor cells with zymogen granules (H and E, ×200); (c) PAS with diastase (PAS-D) stain highlighting the zymogen granules (×200); (d) DOG-1 positive tumor cells (×200).

Histologically, the cases with ETV6 gene rearrangement demonstrated papillary-cystic architecture (Figures 5(a)–5(c)). They were well circumscribed. Tumor cells exhibited clear cytoplasm, were focally vacuolated, and lacked zymogen granules. Cribriform pattern was also noted. Eosinophilic secretions were present in both the cases. Eleven cases with negative ETV6 gene rearrangement demonstrated solid, microcystic and papillary pattern (Figures 6(a)–6(c)). Overt serous distribution characterized by presence of zymogen granules was also noted in 8 of 11 cases. Eosinophilic secretions were present in only 2 cases.

Immunohistochemical results were similar to that noted in cytology cases with tumors with ETV6 gene rearrangement exhibiting positivity for S100, mammaglobin, and GATA-3 and lack of staining for DOG-1. In contrast, all of eleven translocation negative cases were positive for DOG-1 and negative for S100, mammaglobin, and GATA-3. Based on immunohistochemical and translocation findings, revised histologic diagnosis was changed in only two cases from ACCi to SC.

## 4. Discussion

Acinic cell carcinoma is a low-grade salivary gland carcinoma characterized by serous differentiation. Given the recent discovery of SC, a salivary gland carcinoma that mimics ACCi both on histology and on cytology, we undertook a reevaluation of ACCi diagnosed at our institution [1, 4–8]. Based on ETV6 gene rearrangement and immunohistochemical profile, 15.4% of surgically resected parotid gland ACCi and 14.3% of cases with FNA diagnosis of parotid ACC were reclassified as SC. These figures are close to the reclassification rate of 19% previously reported by Bishop et al. in their reevaluated cases that were previously diagnosed as parotid ACC on histology [4].

Similar to prior studies, we found that cytologic and histologic distinction between SC and ACCi is difficult [1, 4–10]. Solid, cystic, microcystic, cribriform pattern, papillary architecture, and vacuolated cells were noted in both tumors. Lack of zymogen granules has been described as one of the features of SC [6]. In our series we found that absence of zymogen granules was noted in SC as well as few cases of ACC. Background secretions were also noted in both tumors. We have too few cases of SC in our series to compare the predominance of morphologic features between the two tumors.

Similar to prior studies, immunohistochemistry proved to be useful in separating both entities [11–13]. In our study, we used an immunohistochemical panel of S100, mammaglobin, GATA-3, and DOG-1 as a means to distinguish SC from ACCi. DOG-1 was consistently positive in all cases of ACCi and negative in SC. All cases of SC demonstrated S100, mammaglobin, and GATA-3 positive tumor cells and these markers were consistently negative in ACC. Skalova noted in her original article that SC has a similar immunohistochemical profile to secretory breast cancer and expresses S-100 protein, mammaglobin, and vimentin [1]. Schwartz et al., in their study on the GATA-3 expression in different salivary gland tumors, noted that GATA-3 staining was observed in most of the salivary gland tumor types and is weakly positive in both acini and ducts of background benign salivary gland tissue, but diffuse immunolabeling was consistently seen in high-grade salivary duct carcinoma and secretory carcinoma, that is, the two tumor types that most closely resemble breast neoplasia [12]. However HER2 gene amplification has only been reported in high-grade salivary duct carcinoma and is absent in SC [14]. Although the immunoprofile of salivary duct carcinoma is similar to that of SC, the former is distinguished from the latter by its high-grade morphology and usually presence of necrosis. DOG-1 immunostain is usually negative in SC and demonstrates intense apical membranous staining around lumina and variable cytoplasmic positivity in most cases of ACCi [13].

The presence of ETV6-NTRK3 fusion gene is specific for SC and has not been shown in any other salivary gland tumor so far [11]. A particularly interesting finding in our study is the one SC case demonstrating ETV6 gene 3′ interstitial deletion with absence of the traditional translocation. This is in accordance with findings Pinto et al. who noted the presence of* ETV6* amplification in one SC case and deletion of this gene in another and suggested the possible presence of alternative pathways involving the* ETV6-NTRK3* gene that could be related to the pathogenesis of SC [15]. In addition, there has been awareness of a number of SC cases positive for the ETV6 gene split as visualized by FISH but in which the classical ETV6-NTRK3 fusion transcript (exon 5-exon 15 junction) was not detected by standard reverse transcriptase-polymerase chain reaction (RT-PCR). Skálová et al. identified 5 SC cases with atypical junctions, exon 4 of ETV6 with exon 14 of NTRK3, and exon 5 of ETV6 with exon 14 of NTRK3 that have not been described in SC so far [16]. They also confirmed the observation of Ito et al. that a subset of SC cases very likely harbors ETV6 fused with non-NTRK genes (ETV6-X fusion) [17]. Their study also suggested that cases with ETV6 gene fusing with genes other than NTRK3 or those cases displaying atypical exon junctions of ETV6-NTRK3 are associated with more aggressive/infiltrative histologic features of SC and less favorable clinical outcomes. Our cases of SC have not shown any recurrence during a follow-up period of up to 2 years.

The differential diagnosis of SC is wide and includes, in addition to zymogen granule poor ACCi, low-grade cribriform cystadenocarcinoma (LGCCC), low-grade mucoepidermoid carcinoma, and cystadenocarcinoma/adenocarcinoma, NOS.

Low-grade cribriform cystadenocarcinoma (LGCCC) is a novel noninvasive low-grade salivary gland adenocarcinoma that was first described as low-grade salivary duct carcinoma and was renamed by the World Health Organization in 2005 [18]. LGCCC expresses p63, vimentin, high molecular-weight cytokeratin, CK 7, CK19, MUC-1, S-100 protein, mammaglobin, and GATA-3 and is negative for MUC-4 and adipophilin [19]. LGCCC shows a complete intact myoepithelial rim around tumor nests and extensive p63 positive basal cell layer, which is not a feature of SC and an important distinctive hallmark [3].

The cobblestone-like appearance and squamous differentiation of low-grade mucoepidermoid carcinoma, in addition to its lack of S100 protein expression, help differentiate this entity from SC. Skalova noted in her study that the HMWK positivity in SC is less intense than is typically seen in MEC and more than 50% of MEC are characterized by a t(11; 19) translocation coding for a CRTC1-MAML2 fusion protein with no evidence so far of any ETV6 alterations [11].

Finally, adenocarcinoma NOS is a diagnosis of exclusion and represents an unclassifiable salivary gland carcinoma. The diagnostic ETV6 alterations help identifying SC cases from adenocarcinomas, NOS.

## 5. Conclusion

Based on our case series, ACCi is infrequently misclassified (13.3% in our case series). Characteristic immunoprofile (S100, mammaglobin, and GATA-3 positivity) and ETV6 gene (12p13) rearrangement may prove useful in identifying cases of SC, thereby preventing their misclassification as ACCi. Similar to the prior study by Bellevicine et al. [9], the presence of ETV6 3′ interstitial deletion in one of our cases suggests that there may be additional ETV6 related genetic alterations contributing to the pathogenesis of SC.

## Figures and Tables

**Table 1:** Summary of demographics, pathologic, and ancillary test findings for patients with original cytology diagnosis of acinic cell carcinoma (ACCi) involving parotid glands.

Case number	Age	Sex	ZG	ETV6	Immunohistochemistry				Revised diagnosis
					S100	Mammaglobin	GATA-3	DOG-1	
1	46	F	Present	Neg.	−	−	−	+	ACCi
2	53	M	Absent	Neg.	−	−	−	+	ACCi
3	54	F	Present	Neg.	−	−	−	+	ACCi
4	69	M	Absent	Neg.	−	−	−	+	ACCi
5	68	M	Present	Neg.	−	−	−	+	ACCi
6	54	M	Absent	Pos.	+	+	+	+	SC
7	11	F	Present	Neg.	−	−	−	+	ACCi
8	18	F	Present	Neg.	−	−	−	+	ACCi

**Table 2:** Summary of demographics, pathologic, and ancillary test findings in 13 patients with surgical diagnosis of acinic cell carcinoma (ACCi) involving parotid gland.

Case number^*∗*^* *	Age	Sex	ETV6	Immunohistochemistry				Revised diagnosis
				S100	Mammaglobin	GATA-3	DOG-1	
1	46	F	Neg.	−	−	−	+	ACCi
2	53	M	Neg.	−	−	−	+	ACCi
3	54	F	Neg.	−	−	−	+	ACCi
4	69	M	Neg.	−	−	−	+	ACCi
5	68	M	Neg.	−	−	−	+	ACCi
6	54	M	Pos.	+	+	+	+	SC
9	33	M	3′ del.	+	+	+	−	SC
10	10	F	Neg.	−	−	−	+	ACCi
11	56	M	Neg.	−	−	−	+	ACCi
12	53	F	Neg.	−	−	−	+	ACCi
13	72	F	Neg.	−	−	−	+	ACCi
14	46	F	Neg.	−	−	−	+	ACCi
15	56	F	Neg.	−	−	−	+	ACCi

^*∗*^Case numbers 1 to 6 represent surgical follow-up of cytology cases 1 to 6 in Table 1.
